# Antimicrobial Resistance and Molecular Investigation of H_2_S-Negative *Salmonella enterica* subsp. *enterica* serovar Choleraesuis Isolates in China

**DOI:** 10.1371/journal.pone.0139115

**Published:** 2015-10-02

**Authors:** Jing Xie, Shengjie Yi, Jiangong Zhu, Peng Li, Beibei Liang, Hao Li, Xiaoxia Yang, Ligui Wang, Rongzhang Hao, Leili Jia, Zhihao Wu, Shaofu Qiu, Hongbin Song

**Affiliations:** 1 Institute of Disease Control and Prevention, Academy of Military Medical Sciences, Beijing, 100071, China; 2 Xiangya Basic Medical College, Central South University, Changsha, 410013, China; 3 Clinical Diagnostic Center, 302 Hospital of PLA, Beijing, China; 4 College of Animal Science and Veterinary Medicine, Shanxi Agricultural University, Jinzhong, 030800, China; Purdue University, UNITED STATES

## Abstract

*Salmonella enterica* subsp. *enterica* serovar Choleraesuis is a highly invasive pathogen of swine that frequently causes serious outbreaks, in particular in Asia, and can also cause severe invasive disease in humans. In this study, 21 *S*. Choleraesuis isolates, detected from 21 patients with diarrhea in China between 2010 and 2011, were found to include 19 H_2_S-negative *S*. Choleraesuis isolates and two H_2_S-positive isolates. This is the first report of H_2_S-negative *S*. Choleraesuis isolated from humans. The majority of H_2_S-negative isolates exhibited high resistance to ampicillin, chloramphenicol, gentamicin, tetracycline, ticarcillin, and trimethoprim-sulfamethoxazole, but only six isolates were resistant to norfloxacin. In contrast, all of the isolates were sensitive to cephalosporins. Fifteen isolates were found to be multidrug resistant. In norfloxacin-resistant isolates, we detected mutations in the *gyrA* and *parC* genes and identified two new mutations in the *parC* gene. Pulsed-field gel electrophoresis (PFGE), multilocus sequence typing (MLST), and clustered regularly interspaced short palindromic repeat (CRISPR) analysis were employed to investigate the genetic relatedness of H_2_S-negative and H_2_S-positive *S*. Choleraesuis isolates. PFGE revealed two groups, with all 19 H_2_S-negative *S*. Choleraesuis isolates belonging to Group I and H_2_S-positive isolates belonging to Group II. By MLST analysis, the H_2_S-negative isolates were all found to belong to ST68 and H_2_S-positive isolates belong to ST145. By CRISPR analysis, no significant differences in CRISPR 1 were detected; however, one H_2_S-negative isolate was found to contain three new spacers in CRISPR 2. All 19 H_2_S-negative isolates also possessed a frame-shift mutation at position 760 of *phsA* gene compared with H_2_S-positive isolates, which may be responsible for the H_2_S-negative phenotype. Moreover, the 19 H_2_S-negative isolates have similar PFGE patterns and same mutation site in the *phs*A gene, these results indicated that these H_2_S-negative isolates may have been prevalent in China. These findings suggested that surveillance should be increased of H_2_S-negative *S*. Choleraesuis in China.

## Introduction

Salmonellosis is recognized as an important public health problem causing human gastroenteritis and bacteremia throughout the world. The spread and infection of this pathogen is caused by the consumption of contaminated food or water in humans and animals [1]. Nontyphoidal *Salmonella* is a major health threat in both developing and developed countries, with an estimated 93.8 million cases and 155,000 deaths each year [2]. *Salmonella enterica* subsp. *enterica* serovar Choleraesuis, which has adapted to swine, primarily causes septicemia, pneumonia and diarrhea [3,4]. This pathogen frequently causes serious outbreaks in pigs in several Asia countries [5]. Moreover, it is highly pathogenic to humans and can cause severe invasive disease, especially in older people with underlying disease [6–8]. *S*. Choleraesuis was reported to be the second most common serovar causing human salmonellosis in Taiwan and the eleventh most common serovar in Thailand [9,10]. Apart from Asia, *S*. Choleraesuis is not often isolated from humans [11]. Contaminated environment, food or water sources could as a reservoir for *S*. Choleraesuis infection of humans.

To identify *Salmonella* and other intestinal bacteria, selective medium such as deoxycholate hydrogen lactose (DHL) or *Salmonella-Shigella* (SS) agar are the conventional methods. As a typical phenotype of *Salmonella*, the production of hydrogen sulfide (H_2_S) is detected through Triple Sugar Iron Agar (TSI). *Salmonella* has been considered to be typical H_2_S-producer, however many serovar of *Salmonella*, such as *S*. Choleraesuis, have been reported to be non-H_2_S-producer recently [12–16].

In this study, we examined 21 *S*. Choleraesuis isolates identified during national surveillance of salmonellosis in China between 2010 and 2011. The majority of these isolates (90%) exhibited a H_2_S-negative phenotype. To our knowledge, human infections caused by H_2_S-negative *S*. Choleraesuis isolates have not previously been reported. The 21 *S*. Choleraesuis isolates were analyzed by antimicrobial susceptibility testing. We also performed pulsed-field gel electrophoresis (PFGE), multilocus sequence typing (MLST), and clustered regularly interspaced short palindromic repeat (CRISPR) analysis to evaluate the molecular characteristics and genetic relationships of these isolates [17–20].

## Materials and Methods

### Ethics statement

The study was approved and authorized for each location by the institutional ethics committees of the Academy of Military Medical Sciences of the Chinese People's Liberation Army, Beijing, China. The institutional review board of the Academy of Military Medical Sciences waived the need for written informed consent from the participants.

### Isolation and identification of *S*. Choleraesuis

Twenty-one presumptively *Salmonella*-like colonies were separated from 21 individual patients with diarrhea in China between 2010 and 2011. These presumptively *Salmonella*-like colonies cultured on SS agar (Land Bridge Technology, Beijing, China) at 37°C for 24 h, and the colorless colonies indicated a H_2_S-negative phenotype. All of the colorless colonies were further identified using API 20E biochemical test kits (bioMerieux SA, Marcy I’Etoile, France) and were serotyped by serological testing (SSI Diagnostic, Hillerod, Denmark) according to standard methods. To confirm H_2_S production, the colorless colonies were subcultured on TSI agar at 37°C for 24 h. All isolates (including two H_2_S-positive and ninteen H_2_S-negative isolates) were used for further analysis.

### Antimicrobial susceptibility testing

The antimicrobial susceptibility of the 21 *S*. Choleraesuis isolates was tested against 21 different antibiotics using broth microdilution in a 96-well microtiter plate (Sensititre; Trek Diagnostic Systems, Thermo Fisher Scientific Inc., Cleveland, OH, USA) and the results were interpreted according to the Clinical and Laboratory Standards Institute guidelines [21]. The antibiotics used in this study included: Cephalosporins: ceftazidime (CAZ), ceftriaxone (CRO), cefepime (FEP), cefoperazone (CFP), cefazolin (CFZ) and cefoxitin (FOX); Fluoroquinolones: levofloxacin (LVX) and norfloxacin (NOR); Penicillins: piperacillin (PIP), ticarcillin (TIC), ampicillin (AMP) and ticarcillin-clavulanic acid (TIM); Aminoglycosides: tobramycin (TOB), gentamicin (GEN) and amikacin (AMK); Phenicols: chloramphenicol (CHL); Sulfonamides: trimethoprim-sulfamethoxazole (SXT); Thienamycins: imipenem (IPM); Nitrofurans: nitrofurantoin (NIT); Tetracyclines: tetracycline (TET) and β-lactams: aztreonam (ATM). *Escherichia coli* ATCC 25922 was used as a control strain. To clarify the molecular mechanism of fluoroquinolone resistance, the *gyrA* and *parC* genes were amplified by PCR using the primers described in S1 Table. The PCR products were sequenced by Sangon Biotech (Shanghai, China) and aligned for analysis.

### Multilocus sequence typing analysis

MLST of the 21 *S*. Choleraesuis isolates was carried out using the protocols described at http://mlst.warwick.ac.uk/mlst/dbs/Senterica. Total DNA of *S*. Choleraesuis was extracted using a TIANamp Bacteria DNA Kit (Tiangen Biotech, Beijing, China) according to the manufacturer’s directions. The primers and PCR conditions used were the same as previously described [15]. The PCR products were sequenced and the DNA sequences were imported into DNAStar version 7.1.0 (Lasergene, Madison, WI, USA) for analysis. Each isolate of *S*. Choleraesuis was uploaded to the MLST database and determined the sequence type (ST). STs were analyzed for clonal relationship using the eBURST version 3 program (http://ebrust.mlst.net) [22].

### Pulsed-field gel electrophoresis analysis

All 21 *S*. Choleraesuis isolates included in this study were analyzed by PFGE according to the standard methods outlined by PulseNet [23]. The DNA was digested by *Xba*I restriction enzyme (Takara, Dalian, Japan) at 37°C for 3 h. Then, electrophoresis of the digested DNA was performed using a CHEF Mapper PFGE system (Bio-Rad, Hercules, CA, USA) using 1% SeaKem agarose in 0.5× Tris-borate-EDTA for 19 h. Macrorestriction patterns were compared using BioNumerics Fingerprinting software version 6.01 (Applied Math, Austin TX, USA). The dendrogram was based on unweighted pair-group method by use of average linkages and pairwise Dice coefficients.

### Clustered regularly interspaced short palindromic repeat analysis

A previously described strategy was used in this analysis [24]. We used forward primer A1 (5’-GTRGTRCGGATAATGCTGCC-3’) and reverse primer A2 (5’-CGTATTCCGGTAGATBTDGATGG-3’) to amplify CRISPR locus 1 and forward primer B1 (5’-GAGGAATACYYTRATCGTTAACGCC-3’) and reverse primer B2 (5’-GTTGCDATAKGTYGRTRGRATGTRG-3’) to amplify CRISPR locus 2. The PCR conditions were 95°C for 5 min, followed by 35 cycles of 94°C for 1 min, 59°C for 1 min, and 72°C for 1 min, followed by an extension period of 10 min at 72°C, then cooling to 4°C. The spacers of CRISPR 1 and CRISPR 2 were identified using CRISPR finder (http://crispr.u-psud.fr/Sever/) [19]. Spacers and direct repeats were identified using the Institut Pasteur CRISPR database for *Salmonella* (http://www.pasteur.fr/recherche/genopole/PF8/crispr/CRISPRDB.html). No matching spacers or direct repeats were assigned according to appropriate spacer nomenclature [24]. We used CRISPRcompar for spacer arrangement analysis and DNAstar version 7.1.0 (Lasergene) for single nucleotide polymorphism analysis [19].

### Mutations within the *phs* operon sequence

The *phs* operon, containing the *phsA*, *phsB*, and *phsC* genes, is essential for the generation of H_2_S [25]. Primers for these three genes were designed using the Primer Premier Software version 6.0 (PREMIER Biosoft, Palo Alto, CA, USA) based on *S*. Choleraesuis strain SC-B67 (NC_006905.1) and are shown in S1 Table. The PCR conditions were 95°C for 5 min, followed by 30 cycles of 94°C for 30 s, 55°C for 45 s, 72°C for 1 min, followed by an extension period of 10 min at 72°C, then cooling to 4°C. Sequence results were imported into DNAStar version 7.1.0 (Lasergene) to assemble the complete operon. We aligned all of the sequences using MEGA software version 5.2 to identify mutations within DNA and protein sequences.

## Results

### Identification of H_2_S-negative *S*. Choleraesuis

During national surveillance for salmonellosis in China between 2010 and 2011, we detected 21 *S*. Choleraesuis isolates from 21 individual patients, including six (29%) from Nanjing and fifteen (71%) from Shanghai. All the isolates were recovered from diarrhea patients. Phenotype analysis revealed that, there were nineteen H_2_S-negative and two H_2_S-positive *S*. Choleraesuis by H_2_S phenotype analysis (S1 Fig and S2 Table).

### Antimicrobial susceptibility testing

Of the 21 *S*. Choleraesuis isolates, two H_2_S-positive *S*. Choleraesuis were sensitive to all of the tested antimicrobials. In the 19 H_2_S-negative *S*. Choleraesuis, most were resistant to chloramphenicol (79%), followed by ampicillin (74%), gentamicin (74%), ticarcillin (74%), trimethoprim-sulfamethoxazole (63%), tetracycline (58%), tobramycin (42%), and piperacillin (26%). Resistance was not detected to amikacin, aztreonam, cefazolin, cefepime, cefoperazone, cefoxitin, ceftazidime, ceftriaxone, imipenem, levofloxacin, or nitrofurantoin. Fifteen (79%) H_2_S-negative *S*. Choleraesuis isolates were multidrug resistant (resistant to three or more classes of antimicrobial agents) (S3 Table).

Moreover, six (32%) of the H_2_S-negative *S*. Choleraesuis isolates were intermediate or resistant to norfloxacin, a fluoroquinolone. We detected mutations in the *gyrA* and *parC* genes resulting in fluoroquinolone resistance. Half of the isolates were only detected single-site mutation in the *parC* gene (Table 1). In the remaining norfloxacin-resistant isolates, double mutation sites in the *gyrA* and *parC* gene were detected at the same time. Among these mutations, position 83 in GyrA protein and position 80 in ParC protein have been reported in most fluoroquinolone-resistant *S*. Choleraesuis isolates [26]. However, positions 78 and 117 in the ParC protein were newly discovered mutation sites in *S*. Choleraesuis and may contribute to intermediate or full resistance to norfloxacin (GenBank accession numbers: KP184386–KP184397). Although these mutations are novel, further work is required to demonstrate whether these mutations contribute to resistance.

**Table 1:** Mutations detected in the *gyrA* and *parC* gene of H_2_S-negative *S*. Choleraesuis isolates.

Strain number	*gyrA* gene	*parC* gene		
	Ser83	Gly78	Ser80	Ala117
SC1209	-	-	-	Ala117Pro
SC1214	-	-	-	Ala117Pro
SC1215	Ser83Tyr	-	Ser80Arg	-
SC1218	Ser83Tyr	Gly78Cys	-	-
SC1219	Ser83Tyr	Gly78Cys	-	-
SC1221	-	Gly78Cys	-	-

Ser, serine. Gly, glycine. Ala, alanine. Tyr, tyrosine. Cys, cysteine. Arg, arginine. Pro, proline.

### MLST analysis

**Fig 1:**
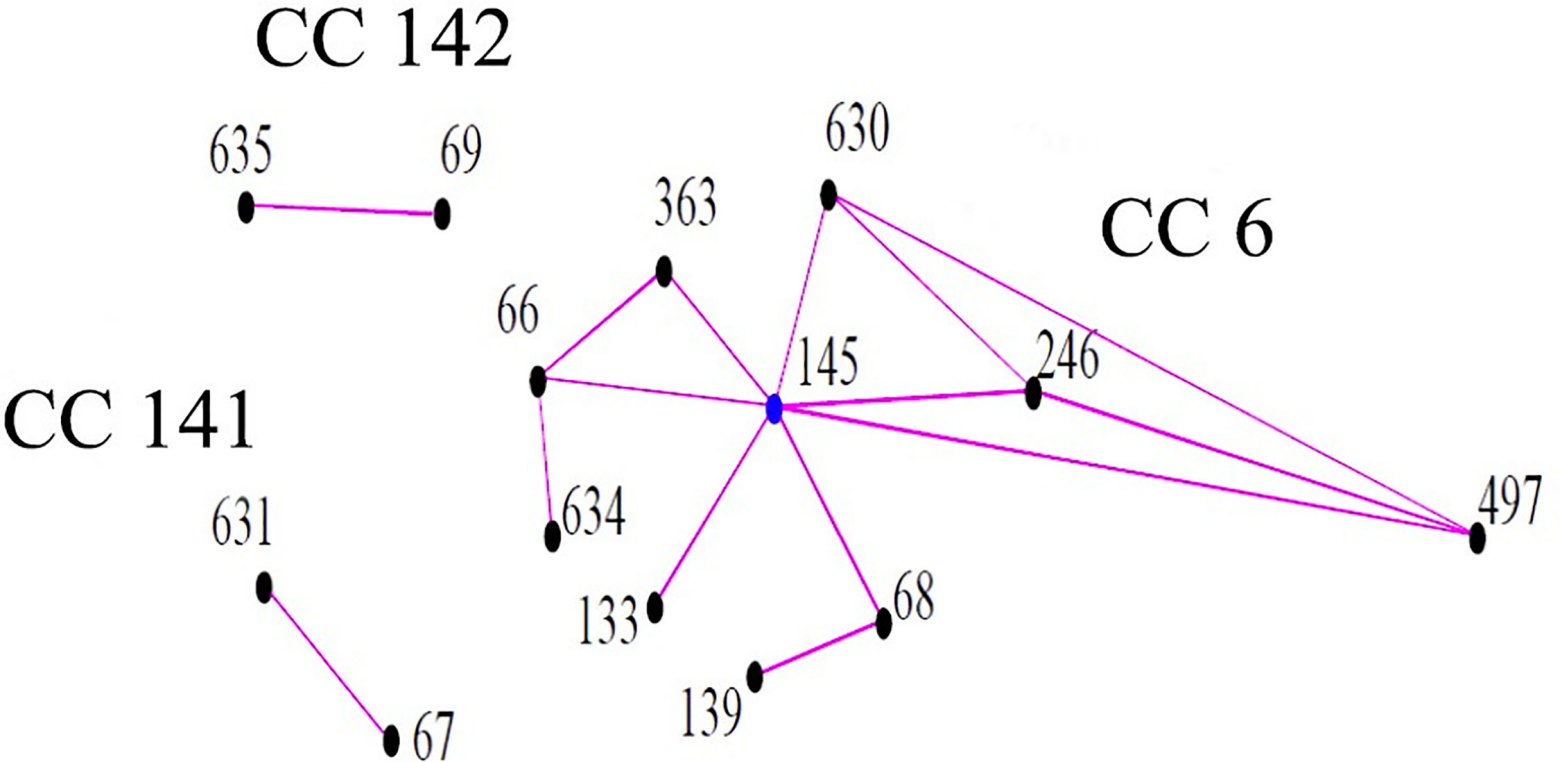
Phylogenetic relationships of all *S*. Choleraesuis STs from MLST database by eBURST analysis. All *S*. Choleraesuis STs were divide into three clonal complexes. The blue solid circle represents the founder clonal complex. The red lines indicate SLVs between STs.

The 21 *S*. Choleraesuis isolates were divided into two MLST STs, designated ST68 and ST145, by eBURST analysis. The two STs identified in this study both belonged to clonal complex 6 (CC6)(Fig 1). ST145 was assigned as the founder of this complex. ST68 was a single locus variant (SLV) of ST145. The H_2_S-negative and H_2_S-positive *S*. Choleraesuis isolates belonged to different STs. All 19 H_2_S-negative isolates belonged to ST68, including all isolates from Shanghai (SC1207–1221) and four isolates from Nanjing (SC1201–1204). The remaining two isolates from Nanjing (SC1205 and SC1206) were typed as ST145. These results suggested that ST68 may be the most prevalent ST in Shanghai and Nanjing.

### Analysis of PFGE patterns

**Fig 2:**
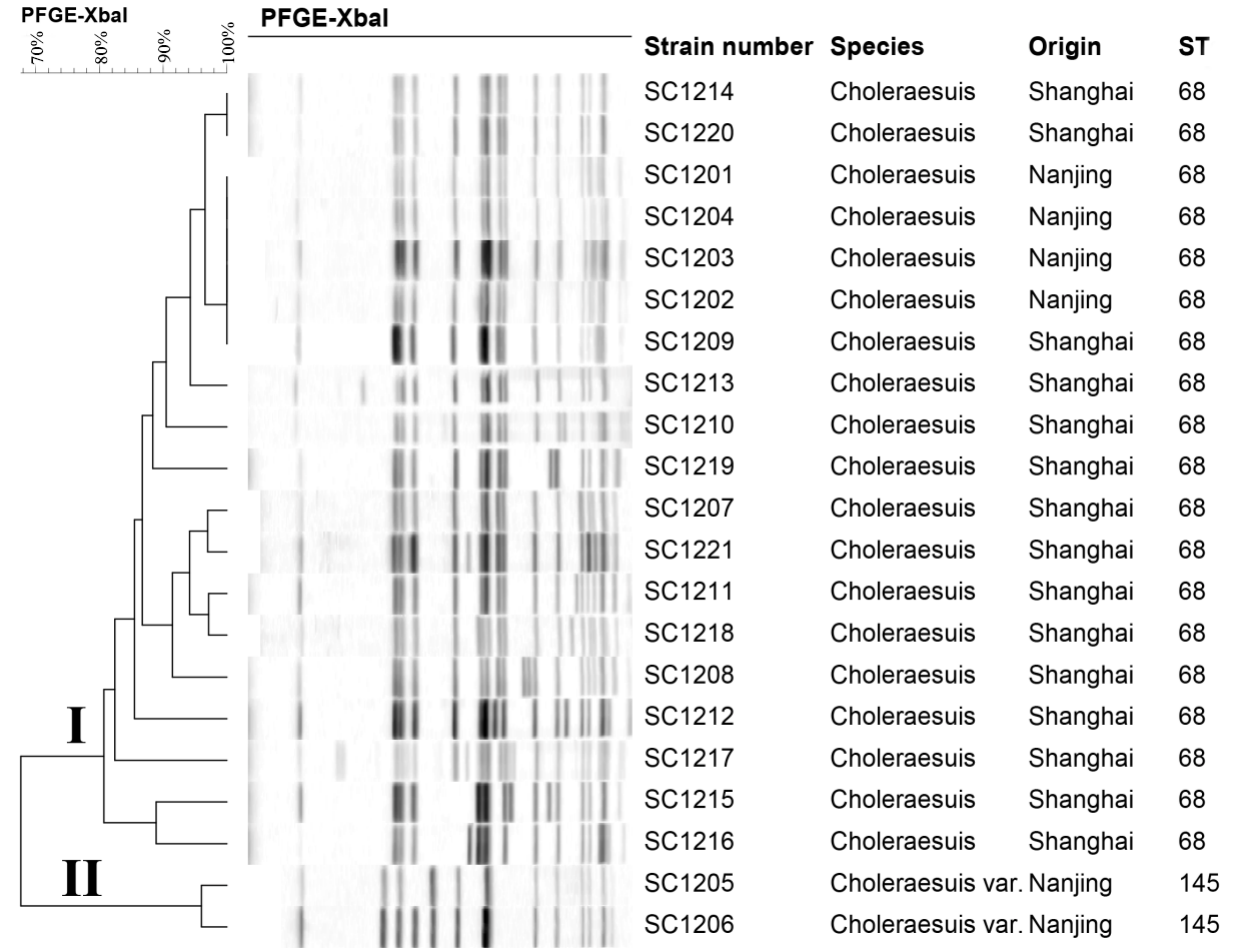
Dendrogram analysis of PFGE for the 21 *S*. Choleraesuis isolates by *Xba*I-digestion. The strain number, species, origin and ST are shown for each isolate.

The 21 *S*. Choleraesuis isolates were subtyped into 16 unique *Xba*I PFGE patterns (Fig 2). The 19 H_2_S-negative isolates produced 14 different profiles, while the two H_2_S-positive isolates produced two profiles. Cluster analysis of the PFGE patterns revealed two main distinct groups accounting for approximately 70% pattern similarity. In Group I, all of the 19 isolates, belonging to ST68, were H_2_S-negative. This group included three isolates from Shanghai (SC1209, SC1214, and SC1220) and four from Nanjing (SC1201–1204) that shared approximately 96% similarity in their PFGE patterns. This finding indicated that H_2_S-negative *S*. Choleraesuis isolates belonging to ST68 have the potential for cross-region dissemination. In Group II, there were only two isolates, which belonging to ST145 were identified as H_2_S-positive, and shared approximately 96% similarity as well.

### CRISPR analysis

All isolates belonging to ST68 were found to possess the same five spacers in CRISPR 1 (Table 2). Two isolates belonging to ST145 (SC1205 and SC1206) had lost spacers STM1 and ParC4 compared with isolates belonging to ST68. In CRISPR 2, all isolates belonging to ST68 shared the same spacers in addition to SC1217. Three new CRISPR spacers were discovered in this isolate, namely spacers CholB87, CholB88, and CholB89. These new spacers did not match any spacers currently in the database. SC1205 and SC1206 shared the same spacers in both CRISPR 1 and CRISPR 2 (GenBank accession numbers: KP184340–184345, 18348–18368, 18371–18385).

**Table 2:** CRISPR spacer content of the 21 *S*. Choleraesuis isolates.

Strain number	ST	CRISPR spacer content	
		CRISPR 1	CRISPR 2
SC1201-1204	68	STM1-Chol1-Chol2-ParC4-Chol3	EntB0-CholB1-CholB2-CholB3-ParCB1-ParCB2-CholB4
SC1205-1206	68	Chol1-Chol2-Chol3	EntB0-CholB1-CholB2-CholB3-ParCB1-ParCB2-CholB4
SC1207-1216	145	STM1-Chol1-Chol2-ParC4-Chol3	CholB1-CholB2-CholB3-CholB4-CholB5-CholB6
SC1217	68	STM1-Chol1-Chol2-ParC4-Chol3	CholB87^#^-CholB3-ParCB1-CholB88^#^-CholB89^#^
SC1218-1221	68	STM1-Chol1-Chol2-ParC4-Chol3	EntB0-CholB1-CholB2-CholB3-ParCB1-ParCB2-CholB4

^#^Novel spacer identified in this study.

### Mutation within the *phs* operon sequence

**Fig 3:**
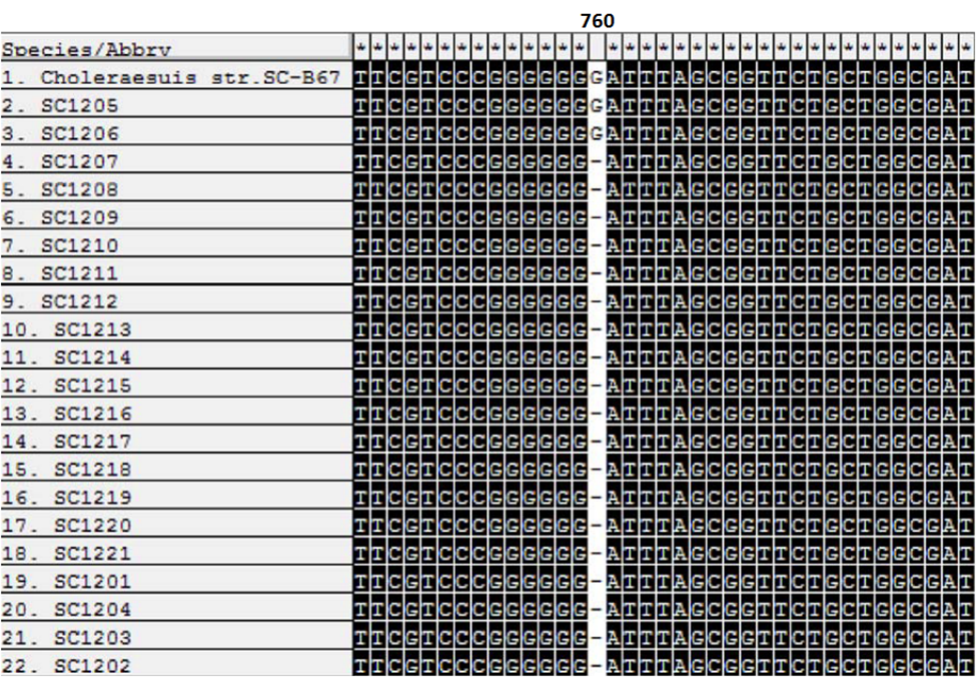
Alignment of *phsA* sequences in 21 *S*. Choleraesuis. The deletion of G at position 760 resulted in a frame-shift mutation. The first sequence is H_2_S-positive *S*. Choleraesuis strain SC-B67 (NC_006905.1).

We analyzed the *phs* operon of all 21 *S*. Choleraesuis isolates and compared it with that of *S*. Choleraesuis strain SC-B67. In all 19 H_2_S-negative isolates, a single base deletion was detected at position 760 in the *phsA* gene (Fig 3). This deletion led to a frame-shift mutation, resulting in a nonfunctional protein that may be unable to generate H_2_S. No mutations in the *phsB* and *phsC* genes were detected (data not shown). These results suggested that the H_2_S-negative phenotype may result of the frame-shift mutation at position 760 in the *phsA* gene (GenBank accession numbers: KP184398–184416, 18419–18420).

## Discussion

The incidence of H_2_S-negative *Salmonella* isolates identified from chicken, pork, seafood, and humans has been reported globally in recent years [12–16]. In China, 40% of the isolates in retail meat were H_2_S-negative *Salmonella*, including *S*. Derby, *S*. Heidelberg, *S*. Rosenthal, *S*. Enteritidis, *S*. Indiana, *S*. Typhimurium, *S*. London, and *S*. Virchow [16]. In Japan, researchers detected H_2_S-negative *S*. Typhimurium and *S*. Infantis strains in retail chicken meat and found nonsense mutations at positions 1440 and 358, respectively, in the *phsA* gene [13]. We previously reported 17 H_2_S-negative *S*. Senftenberg isolates worldwide with a nonsense mutation at position 1621 of the *phsA* gene [15]. Here, we identified 19 H_2_S-negative isolates among 21 *S*. Choleraesuis isolates detected in China during the period from 2010 to 2011. A frame-shift mutation at position 760 of the *phsA* gene was detected in all 19 H_2_S-negative *S*. Choleraesuis isolates, indicating that *phsA* gene maybe unstable and is likely responsible for the H_2_S-negative phenotype. It has been shown that the accumulation of thiosulfate (S_2_O_3_^2–^), which was unable to be converted to H_2_S by H_2_S-negative *S*. *enterica* in the intestines, could promote *S*. Typhimurium survival and inflammation due to tetrathionate (S_2_O_3_^–^) respiration by reducing thiosulfate (S_2_O_3_^2–^) consumption [27–30]. Thus, we propose that H_2_S-negative *S*. Choleraesuis may possess growth advantages when competing with other microbes in the lumen and may have stronger survival abilities compared with H_2_S-positive isolates. Furthermore, the incidence of infections caused by H_2_S-negative *Salmonella* isolates may have been greatly underestimated because of their atypical biochemical characteristics and the methods of detection employed. Taken together, the appearance of H_2_S-negative isolates may present an even greater challenge in the surveillance and control of *Salmonella*.

The 19 H_2_S-negative *S*. Choleraesuis isolates analyzed in this study all belonged to ST68, which is a single locus variant of ST145, and shared some spacers with the isolates belonging to ST145 in the CRISPR 1 and 2 loci. These results indicated that the H_2_S-negative isolates may be derived from isolates belonging to ST145. The 19 H_2_S-negative isolates were also found to share similar PFGE profiles and possessed the same mutation in the *phsA* gene. These data provide evidence that the 19 H_2_S-negative *S*. Choleraesuis isolates are closely related genetically and may have developed into a new subgroup in Eastern China. Moreover, three H_2_S-negative isolates from Shanghai shared similar PFGE patterns with four H_2_S-negative isolates from Nanjing, with approximately 96% similarity in Group I. This suggested that H_2_S-negative *S*. Choleraesuis may disseminate across regions and may have been prevalent in Shanghai and Nanjing. Therefore, more attention should be paid to avoid further dissemination of H_2_S-negative *S*. Choleraesuis isolates in China.

Fifteen (79%) of the H_2_S-negative *S*. Choleraesuis isolates were multidrug resistant and most of the isolates exhibited high resistance to conventional antibiotics, such as chloramphenicol, ampicillin, gentamicin, tetracycline, and trimethoprim-sulfamethoxazole. Among the H_2_S-positive *S*. Choleraesuis isolated from humans have been reported, the prevalence of multidrug resistance was 76% in Taiwan and nearly 90% in Thailand [31,32].Compared with these reported isolates, the H_2_S-negative *S*. Choleraesuis isolates detected in this study displayed high rates of multidrug resistance as well. In Taiwan, most H_2_S-positive isolates (64%) exhibited resistance to the fluoroquinolone, norfloxacin [31]. In our study, only six (32%) of H_2_S-negative isolates were found to possess intermediate or full resistance to norfloxacin. One mechanism of fluoroquinolone resistance was reported to be associated with the presence of multiple mutations in the gyrase genes (*gyrA* and *gyrB*) and the topoisomerase IV genes (*parC* and *parE*) [33–36]. It has been reported that mutations at positions 83 in GyrA and position 80 in ParC were present in most fluoroquinolone-resistant *S*. Choleraesuis isolates [26,37–40]. Among the mutations detected in this study, mutations Ser83Tyr in GyrA and Ser80Arg in ParC have already been reported; however, two mutations Gly78Cys and Ala117Pro in the ParC protein have not previously been reported. In addition, we found that the norfloxacin-resistant isolates had mutations both in the *gyrA* and *parC* genes simultaneously, indicating that the instability of these genes may enhance resistance to norfloxacin. Moreover, cephalosporin-resistant isolates have frequently been detected in *S*. Choleraesuis and may therefore pose another threat to public health [32,41,42]. However, of the six cephalosporin antibiotics tested in our study, no cephalosporin-resistant isolates were detected. A previous study reported that when endogenous H_2_S was suppressed, bacteria could increased the sensitivity to a multitude of antibiotics [43]. These results suggested that bacterial H_2_S is cytoprotective against antibiotics. This phenomenon could explain our finding that all 19 H_2_S-negative *S*. Choleraesuis isolates exhibited susceptibility to cephalosporins.

## Conclusion

In this study, a high proportion of H_2_S-negative *S*. Choleraesuis were detected in Eastern China. These isolates, which display a high prevalence of multidrug resistance, may pose a new threat to public health in the future. In response, we should strengthen surveillance for the appearance and spread of H_2_S-negative *Salmonella* isolates in China and throughout the world.

## Supporting Information

S1 Fig Phenotype analysis between H_2_S-positive and H_2_S-negative *S*. Choleraesuis isolates on TSI agar (A) and SS agar (B). H_2_S-positive *S*. Choleraesuis exhibited the black colony, while the H_2_S-negative *S*. Choleraesuis exhibited the colorless colony.
(PDF)

S1 Table Primer sequences used for PCR amplifition of the *gyrA*, *parC*, *phsA*, *phsB* and *phsC* gene. (PDF)

S2 Table Related information of 21 *S*. Choleraesuis isolates detected during national surveillance of salmonellosis. (PDF)

S3 Table Antimicrobial susceptibility of *S*. Choleraesuis isolates from humans. (PDF)
