# Supplementary material for: Antimicrobial Resistance and Molecular Investigation of H2S-Negative Salmonella enterica subsp. enterica serovar Choleraesuis Isolates in China
Source: PLoS One. 2015 Oct 2;10(10):e0139115. doi: 10.1371/journal.pone.0139115 (PMC4592067; doi:10.1371/journal.pone.0139115)
Supplement: S1 Fig — H2S-positive S. Choleraesuis exhibited the black colony, while the H2S-negative S. Choleraesuis exhibited the colorless colony. (PDF) [file pone.0139115.s001.pdf]

A **B**

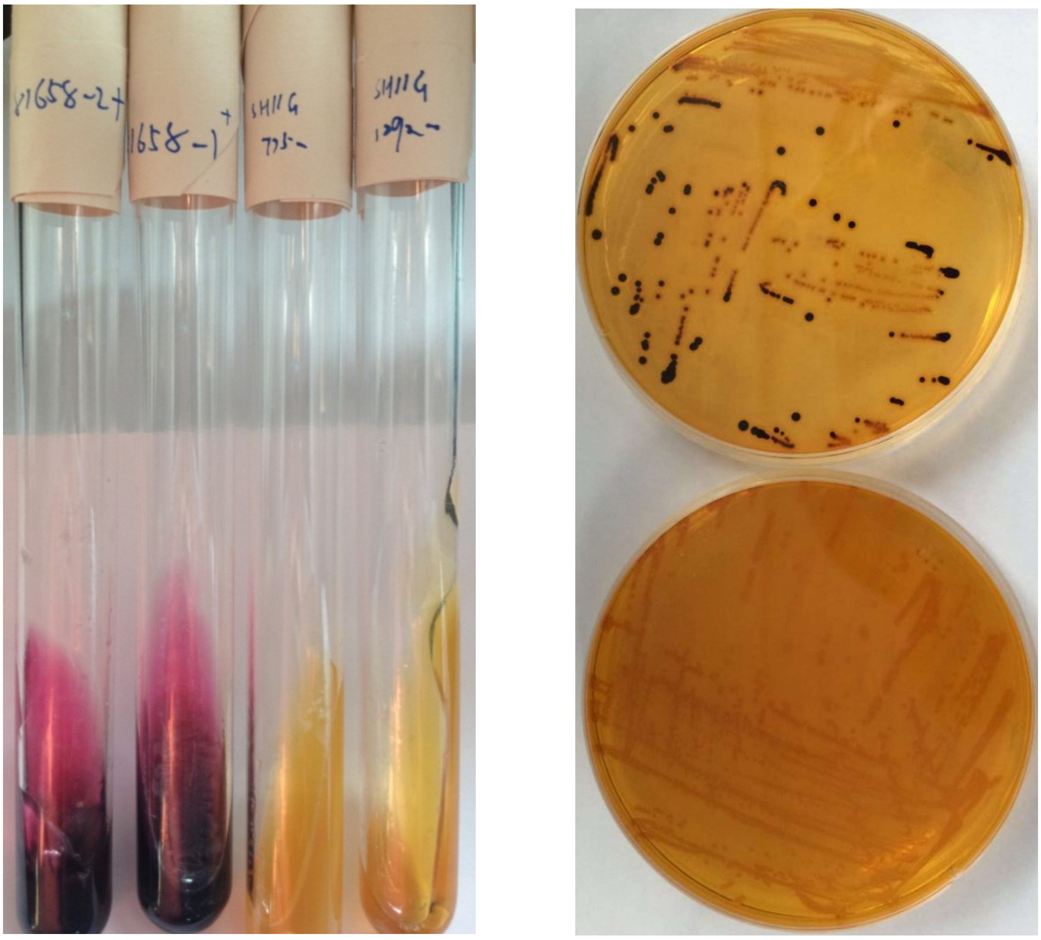

S1 Fig. Phenotype analysis between H_2S-positive and H_2S-negative *S.* Choleraesuis isolates on TSI agar (A) and SS agar (B). H_2S-positive *S.* Choleraesuis exhibited the black colony, while the H_2S-negative *S.* Choleraesuis exhibited the colorless colony.
