# Supplementary material for: Antimicrobial Resistance and Molecular Investigation of H2S-Negative Salmonella enterica subsp. enterica serovar Choleraesuis Isolates in China
Source: PLoS One. 2015 Oct 2;10(10):e0139115. doi: 10.1371/journal.pone.0139115 (PMC4592067; doi:10.1371/journal.pone.0139115)
Supplement: S1 Table — (PDF) [file pone.0139115.s002.pdf]

S1 Table. Primer sequences used for PCR amplifition of the *gyrA, parC, phsA, phsB* and *phsC* gene.

Gene	Forward primers (5'-3')	Reverse primers (5'-3')	Amplicon size (bp)	Position on LT2 chromosome
gyrA	TTATGCGATGTCGGTCATTGTT	TTCACCAGCTCGGCGATT	777	2375481-2376258
parC	CGTGCGTTGCCGTTTATTG	CAACTGATCCAGCGTCGTT	534	3338569-3339103
phsA1	CGTTGGATGCCTGTTCAG	AGGTCGTAGAGCCGATTG	938	2138504-2139479
phsA2	CGCCGTTCAACTGATAGA	AATGGTGAGCTTCGATCC	959	2139310-2140306
phsA3	CATCGTAGAGCTGTTCATCA	CATGTGCGTGTTCAGGAA	975	2140135-2141149
phsB	CAAGCATGAGCAGCACCAC	ATGAGGGAGGAGGGAACCAT	687	2137992-2138720
phsC	GATGGTCTCTATTTGCCGTTCT	GGTGCTGCTCATGCTTGTT	803	2137206-2138051
