# Supplementary material for: Antimicrobial Resistance and Molecular Investigation of H2S-Negative Salmonella enterica subsp. enterica serovar Choleraesuis Isolates in China
Source: PLoS One. 2015 Oct 2;10(10):e0139115. doi: 10.1371/journal.pone.0139115 (PMC4592067; doi:10.1371/journal.pone.0139115)
Supplement: S2 Table — (PDF) [file pone.0139115.s003.pdf]

**S2 Table. Related information of 21 *S.* Choleraesuis isolates detected during national surveillance of salmonellosis.**

| Strain number | Year | Serotype | $H_2S$ phenotype |
|---|---|---|---|
| SC1201 | 2010 | *S.* Choleraesuis | (-) |
| SC1202 | 2010 | *S.* Choleraesuis | (-) |
| SC1203 | 2010 | *S.* Choleraesuis | (-) |
| SC1204 | 2010 | *S.* Choleraesuis | (-) |
| SC1205 | 2011 | *S.* Choleraesuis var. Kunzendorf | (+) |
| SC1206 | 2011 | *S.* Choleraesuis var. Kunzendorf | (+) |
| SC1207 | 2010 | *S.* Choleraesuis | (-) |
| SC1208 | 2010 | *S.* Choleraesuis | (-) |
| SC1209 | 2010 | *S.* Choleraesuis | (-) |
| SC1210 | 2010 | *S.* Choleraesuis | (-) |
| SC1211 | 2010 | *S.* Choleraesuis | (-) |
| SC1212 | 2010 | *S.* Choleraesuis | (-) |
| SC1213 | 2011 | *S.* Choleraesuis | (-) |
| SC1214 | 2011 | *S.* Choleraesuis | (-) |
| SC1215 | 2011 | *S.* Choleraesuis | (-) |
| SC1216 | 2011 | *S.* Choleraesuis | (-) |
| SC1217 | 2011 | *S.* Choleraesuis | (-) |
| SC1218 | 2011 | *S.* Choleraesuis | (-) |
| SC1219 | 2011 | *S.* Choleraesuis | (-) |

| | | | |
|---|---|---|---|
| SC1220 | 2011 | *S.* Choleraesuis | (-) |
| SC1221 | 2011 | *S.* Choleraesuis | (-) |
