# Supplementary material for: Antimicrobial Resistance and Molecular Investigation of H2S-Negative Salmonella enterica subsp. enterica serovar Choleraesuis Isolates in China
Source: PLoS One. 2015 Oct 2;10(10):e0139115. doi: 10.1371/journal.pone.0139115 (PMC4592067; doi:10.1371/journal.pone.0139115)
Supplement: S3 Table — (PDF) [file pone.0139115.s004.pdf]

S3 Table. Antimicrobial susceptibility of *S.* Choleraesuis isolates from humans.

Strain number	Antimicrobial drugs (MIC, μg/ml)								
	**ceftazidime**	**ceftriaxone**	**imipenem**	**nitrofurantoin**	**piperacillin**	**tetracycline**	**cefepime**	**cefoperazone**	**cefazolin**
**SC1201**	S(<1)	S(<1)	S(<4)	S(<32)	I(=64)	R(>8)	S(<8)	S(<16)	S(<8)
**SC1202**	S(<1)	S(<1)	S(<4)	S(<32)	R(>64)	R(>8)	S(<8)	S(<16)	S(<8)
**SC1203**	S(<1)	S(<1)	S(<4)	S(<32)	R(>64)	R(>8)	S(<8)	S(<16)	S(<8)
**SC1204**	S(<1)	S(<1)	S(<4)	S(<32)	R(>64)	R(>8)	S(<8)	S(<16)	S(<8)
**SC1205**	S(<1)	S(<1)	S(<4)	S(<32)	S(<16)	S(<4)	S(<8)	S(<16)	S(<8)
**SC1206**	S(<1)	S(<1)	S(<4)	S(<32)	S(<16)	S(<4)	S(<8)	S(<16)	S(<8)
**SC1207**	S(<1)	S(<1)	S(<4)	S(<32)	S(<16)	S(<4)	S(<8)	S(<16)	S(<8)
**SC1208**	S(<1)	S(<1)	S(<4)	S(<32)	S(<16)	R(>8)	S(<8)	S(<16)	S(<8)
**SC1209**	S(<1)	S(<1)	S(<4)	S(<32)	S(<16)	S(<4)	S(<8)	S(<16)	S(<8)
**SC1210**	S(<1)	S(<1)	S(<4)	S(<32)	R(>64)	R(>8)	S(<8)	S(<16)	S(<8)
**SC1211**	S(<1)	S(<1)	S(<4)	S(<32)	I(=64)	R(>8)	S(<8)	S(<16)	S(<8)
**SC1212**	S(<1)	S(<1)	S(<4)	S(<32)	I(=64)	R(>8)	S(<8)	S(<16)	S(<8)
**SC1213**	S(<1)	S(<1)	S(<4)	S(<32)	S(<16)	S(<4)	S(<8)	S(<16)	S(<8)
**SC1214**	S(<1)	S(<1)	S(<4)	S(<32)	S(<16)	S(<4)	S(<8)	S(<16)	S(<8)
**SC1215**	S(<1)	S(<1)	S(<4)	S(<32)	I(=64)	S(<4)	S(<8)	S(<16)	S(<8)
**SC1216**	S(<1)	S(<1)	S(<4)	S(<32)	R(>64)	R(>8)	S(<8)	S(<16)	S(<8)
**SC1217**	S(<1)	S(<1)	S(<4)	S(<32)	S(<16)	R(>8)	S(<8)	S(<16)	S(<8)
**SC1218**	S(<1)	S(<1)	S(<4)	S(<32)	S(<16)	R(>8)	S(<8)	S(<16)	S(<8)
**SC1219**	S(<1)	S(<1)	S(<4)	S(<32)	S(<16)	S(<4)	S(<8)	S(<16)	S(<8)
**SC1220**	S(<1)	S(<1)	S(<4)	S(<32)	I(=64)	S(<4)	S(<8)	S(<16)	S(<8)
**SC1221**	S(<1)	S(<1)	S(<4)	S(<32)	I(=64)	S(<4)	S(<8)	S(<16)	S(<8)

R, resistant: I: intermediate; S: susceptible.

S3 Table. Antimicrobial susceptibility of *S.* Choleraesuis isolates from humans.

Strain number	Antimicrobial drugs (MIC, μg/ml)							
	**cefoxitin**	**tobramycin**	**levofloxacin**	**gentamicin**	**ticarcillin**	**ticarcillin-clavulanic acid**	**aztreonam**	**ampicillin**
**SC1201**	S(<8)	S(<4)	S(<2)	R(>8)	R(>64)	S(<16)	S(<1)	R(>16)
**SC1202**	S(<8)	S(<4)	S(<2)	R(>8)	R(>64)	I(=64)	S(<1)	R(>16)
**SC1203**	S(<8)	S(<4)	S(<2)	R(>8)	R(>64)	I(=64)	S(<1)	R(>16)
**SC1204**	S(<8)	I(=8)	S(<2)	R(>8)	R(>64)	I(=64)	S(<1)	R(>16)
**SC1205**	S(<8)	S(<4)	S(<2)	S(<4)	S(<16)	S(<16)	S(<1)	S(<8)
**SC1206**	S(<8)	S(<4)	S(<2)	S(<4)	S(<16)	S(<16)	S(<1)	S(<8)
**SC1207**	S(<8)	R(>8)	S(<2)	R(>8)	S(<16)	S(<16)	S(<1)	S(<8)
**SC1208**	S(<8)	R(>8)	S(<2)	R(>8)	R(>64)	S(<16)	S(<1)	R(>16)
**SC1209**	S(<8)	S(<4)	S(<2)	S(<4)	S(<16)	S(<16)	S(<1)	S(<8)
**SC1210**	S(<8)	S(<4)	S(<2)	R(>8)	R(>64)	I(=64)	S(<1)	R(>16)
**SC1211**	S(<8)	R(>8)	S(<2)	R(>8)	R(>64)	I(=64)	S(<1)	R(>16)
**SC1212**	S(<8)	R(>8)	S(<2)	R(>8)	R(>64)	I(=64)	S(<1)	R(>16)
**SC1213**	S(<8)	S(<4)	S(<2)	S(<4)	S(<16)	S(<16)	S(<1)	S(<8)
**SC1214**	S(<8)	S(<4)	S(<2)	S(<4)	S(<16)	S(<16)	S(<1)	S(<8)
**SC1215**	S(<8)	R(>8)	S(<2)	S(<4)	R(>64)	S(<16)	S(<1)	R(>16)
**SC1216**	S(<8)	S(<4)	S(<2)	R(>8)	R(>64)	I(=64)	S(<1)	R(>16)
**SC1217**	S(<8)	S(<4)	S(<2)	R(>8)	R(>64)	S(<16)	S(<1)	R(>16)
**SC1218**	S(<8)	R(>8)	S(<2)	R(>8)	S(<16)	S(<16)	S(<1)	S(<8)
**SC1219**	S(<8)	R(>8)	S(<2)	R(>8)	R(>64)	S(<16)	S(<1)	R(>16)
**SC1220**	S(<8)	R(>8)	S(<2)	R(>8)	R(>64)	I(=64)	S(<1)	R(>16)
**SC1221**	S(<8)	I(=8)	S(<2)	S(<4)	R(>64)	I(=64)	S(<1)	R(>16)

R, resistant: I: intermediate; S: susceptible.

S3 Table. Antimicrobial susceptibility of *S.* Choleraesuis isolates from humans.

Strain number	Antimicrobial drugs (MIC, µg/ml)			
	**chloramphenicol**	**trimethoprim-sulfamethoxazole**	**norfloxacin**	**amikacin**
**SC1201**	R(>16)	R(>2)	S(<4)	S(<16)
**SC1202**	R(>16)	R(>2)	S(<4)	S(<16)
**SC1203**	R(>16)	R(>2)	S(<4)	S(<16)
**SC1204**	R(>16)	R(>2)	S(<4)	S(<16)
**SC1205**	S(<8)	S(<2)	S(<4)	S(<16)
**SC1206**	S(<8)	S(<2)	S(<4)	S(<16)
**SC1207**	R(>16)	S(<2)	S(<4)	S(<16)
**SC1208**	R(>16)	S(<2)	S(<4)	S(<16)
**SC1209**	S(<8)	S(<2)	I(=8)	S(<16)
**SC1210**	R(>16)	R(>2)	S(<4)	S(<16)
**SC1211**	R(>16)	S(<2)	S(<4)	S(<16)
**SC1212**	R(>16)	R(>2)	S(<4)	S(<16)
**SC1213**	S(<8)	S(<2)	S(<4)	S(<16)
**SC1214**	S(<8)	S(<2)	I(=8)	S(<16)
**SC1215**	R(>16)	R(>2)	R(>8)	S(<16)
**SC1216**	R(>16)	R(>2)	S(<4)	S(<16)
**SC1217**	S(<8)	R(>2)	S(<4)	S(<16)
**SC1218**	R(>16)	R(>2)	I(=8)	S(<16)
**SC1219**	R(>16)	R(>2)	R(>8)	S(<16)
**SC1220**	R(>16)	S(<2)	S(<4)	S(<16)
**SC1221**	R(>16)	R(>2)	R(>8)	S(<16)

R, resistant: I: intermediate; S: susceptible.
